# Negotiation of the use of medical contraception: Levers and obstacles within married couples in Benin

**DOI:** 10.1371/journal.pone.0253438

**Published:** 2021-07-22

**Authors:** Togla Aymard Aguessivognon

**Affiliations:** Health and Society Institute, Faculty of Human Sciences, University of Quebec in Montreal, Montreal, Canada; University of Western Australia, AUSTRALIA

## Abstract

In developing countries, millions of married women who want to use medical contraception are unable to do so for various reasons. To address this gap in access to contraception international development actors are emphasizing, among other things, the implementation of empowerment programs for women to enable them to take ownership of issues related to their sexual and reproductive health. Nevertheless, studies show that beyond their socio-demographic characteristics, negotiating contraception as a couple is the essential determinant of medical contraception usage among married women in developing countries. Thus, some authors suggest that this aspect be considered in the strategies of national family planning programs. However, we do not know much about the reasons underlying the negotiation or silence around contraception in Beninese married couples. To fill this gap, we conducted semi-structured interviews with women and men living as married couples in Benin. The results show that this type of negotiation is mainly influenced by specific factors that can act as levers or obstacles. These data could help family planning service providers in Benin and possibly other developing countries to ensure greater contraceptive use among married women.

## Introduction

This research focuses on the negotiation of medical contraception within married couples. By medical contraception, I mean all the products of so-called modern medicine used to prevent the occurrence of an unwanted pregnancy. Thus, medical contraception corresponds to what is referred to in the literature as "modern contraception" while there are only two types of contraception in real life: medical contraception and natural contraception. Because a thing is said to be modern if it is current and fashionable. However, medical, and natural methods are both currently used in the world. They are therefore all modern. For this, the terms “modern contraception” and “traditional contraception” are inappropriate. For example, interrupted coitus is classified as a traditional contraceptive method because it is one of the oldest methods while the contraceptive pill is still called a modern method. However, the contraceptive pill was developed in the mid-1950s. So, it is not so new, and it is not normal that in 2100, the authors continue to classify this medicine among the so-called modern methods, recent, even if this is what is likely to arrive. In short, the so-called modern contraception is medical contraception as opposed to natural contraception.

Beyond this terminological controversy, contraceptive practice is arguably one of the best tools for human development [1]. It promotes female autonomy and equality between the sexes, allows couples to control their fertility and devote themselves to productive activities, reduces poverty and contributes to the well-being of families and improves maternal and child health [2–4]. However, in low- and middle-income countries, 24% of married women aged 15–49 who want to avoid pregnancy express an unmet need for modern contraception [5]. A study reports that this situation is explained by the fear of the deleterious effects of contraceptives, irregular sexual intercourse or sexual abstinence, the low perception of the risk of pregnancy in lactating women or women with postpartum amenorrhea and the opposition of women or their relatives to contraception [6]. Other authors point out that factors such as the age difference between spouses, decision-making power in the couple, attitudes of health service providers, cultural beliefs, etc. influence the contraceptive use of women living in union in developing countries [7–10]. It is also known that women who enjoy financial autonomy use contraception more than those who are poor [11]. To this aspect, the International Conference on Population and Development held in Cairo in 1994 had, among other things, recommended that the public authorities implement policies for the economic empowerment of women that allow them, as well as couples, to access and use more the means of controlling births [12]. Women’s financial independence, however, is not a sufficient condition for contraceptive use in developing countries. In Pakistan, a study highlights that, other things being equal, the couple’s joint decision to use contraception is the most important determinant of medical contraceptive use among married women [13]. Other authors have also found in developing regions that couples who discuss medical contraception are more likely to use it than those who do not; and that the constructive communication of men within the couple favors the medical contraceptive use by their wives [14, 15]. However, Beninese’s spouses do not discuss contraception much [16]. According to the 2012 demographic and health survey (DHS), 90% of Beninese’s women aged 15–49 living in married couples are informed about medical contraception. Yet, the study shows that only 14% of them discussed contraception with another person in the months preceding the survey. And less than 25% of those who had discussed contraception had done so directly with their spouses. Overall, the 2012 DHS results show that medical contraception is not an important topic of discussion among Beninese married couples. Moreover, the 2018 DHS shows that 33% of Beninese’s women aged 15–49 living in union have unmet needs for medical contraception while only 12% of them use it [17]. Despite identifying the discussion of contraception between spouses as the most predictive factor of the use of medical contraceptives among married women living in developing regions [13–15] and researcher suggestions that marital discussion should be considered in the provision of medical contraception services to strengthen the effectiveness of family planning programs in these regions, there is hardly any data on the determinants of the negotiation of the use of medical contraception within married couples in Benin.

This study highlights the factors that influence the dialogue between spouses around medical contraception. The aim is to help and/or encourage family planning service providers to promote negotiation of the use of medical contraception within couple and ultimately strengthen the practice among married women in Benin and other developing countries.

## Methods

### Research approach

To achieve the above objective, a qualitative phenomenological study was carried out. Indeed, this type of research makes it possible to create knowledge from the contents of the consciousness and experiences lived by the people surveyed [18–20].

### Participants: Profiles and selection mode

This study was carried out with volunteers who are women and men aged 18 and over and living as a part of married couple at the time of the study. Each participant was the only member of her/his couple recruited and her/his partner was not informed of the study. Thus, the conjugal partners of the people included in the study were systematically excluded. This measure made it possible to strengthen women’s confidence and freedom of speech given the influence of the patriarchal context weighing on the self-determination of women in Benin, particularly in matters of fertility. To get in touch with potential participants, we combined the snowball strategy with help from health workers working in primary health centers. These health workers are midwives and nurses working in the maternity wards of peripheral health centres and community health workers who support contraceptive services in their villages or city districts. In health centres, midwives and nurses described the study to married people who came for consultation, while community health workers talked about it in their localities and encouraged people to talk to others about the study as well. Thus, interested potential participants contacted health workers who put them in contact with the researcher.

The collection of data from these participants made it possible to reach the theoretical saturation which, according to Pires (1997), occurs when the collection of information no longer reveals new elements making it possible to shed light on the object studied [21]. In addition, it should be noted that the volunteers presented a good sociological representation of the socio-cultural categories / statuses of women and men present in Beninese households, i.e., literate, and illiterate people, those who live in polygamous and monogamous households, people approving and disapproving of medical contraception, those who practice Christianity, Islam and voodoo, people living in rural and urban areas etc. A total of 30 persons were recruited. Their main characteristics are presented in the following Table 1.

**Table 1:** Distribution of respondents according to their socio-demographic characteristics.

Characteristics		Sex	
		Women	Men
Number of respondents (30)		**21**	**9**
Place of residence	Urban	14	5
	Rural	7	4
Household type	polygamous	5	4
	monogamous	16	5
Attitude towards medical contraception	Rejection	14	7
	Approval	7	2
Number of children	≥ 4 children	11	7
	< 4 children	10	2
Level of study	No education and primary level	11	4
	Secondary	6	2
	University	4	3
Religion	Christianity	12	7
	Islam	2	0
	Vodoun	7	2
Age	18–29 years	6	5
	30–39 years	11	1
	≥ 40 years	4	3

Before the recruitment of the 30 participants, the socio-demographic data questionnaire and the interview guide were tested with half a dozen people who were recruited and treated with the same ethical rigor as the participants. The imbalance between the number of participant women and men is not intended the plan was to recruit as many women as there were men. But it turned out that during the recruitment there were fewer men who expressed a willingness to share their experiences in this study. It is the same for people of Muslim faith. Each volunteer was given a pseudonym. In this article, however, those who have been quoted verbatim are simply referred to as woman or man with a minimum of socio-demographic characteristics to ensure confidentiality. On the other hand, it should be noted that are those excluded from the study included people who were not married and those who were married and were having difficulty having a child. This research received ethical clearance (approval certificate number 2138) from the Research Ethics Board for Student Projects Involving Humans (CERPE4)–an institutional ethics committee of the Faculty of Human Sciences of the University of Quebec in Montreal, accessible from the link https://cerpe.uqam.ca/les-comites/cerpe-4/. In Benin, it was impossible to obtain local ethical approval at the time of data collection because the national ethics committee for health research was no longer receiving files due to internal difficulties. The ethics committee of the Institute of Applied Biomedical Sciences was also not operational because its members were being renewed and the new ones had not yet been installed. Faced with this difficulty, an authorization was requested and obtained to collect data based on the submission to and examination of a file on the research project by the Department of Mother and Child Health in the Ministry of Health.

The consent of the participants was obtained in one of two ways. Literate participants gave their written consent after reading the consent form and asking questions. Illiterate participants gave recorded oral consent after being informed in the language they understood of the content of the consent form. All participants were informed of their right to ask questions about the study and to withdraw at any time without prejudice and justification. Answers were provided to any question or concern raised by participants.

### Data: Collection, processing and methods of analysis

Data were collected with support a research assistant who had very good experience in data collection. The research assistant, a woman collected the data from the women and the author, male, collected data from the men. This measure was intended to give confidence to women who might be embarrassed to discuss the subject of research with a man they did not know. Similarly for the men. The research assistant signed a confidentiality agreement and completed the Tri-Council Policy Statement 2 (2018) online training on the ethics of research involving humans.

Data was collected using semi-structured one-on-one interviews in French and the local language *Fon*. Data were recorded on digital audio. The following themes were discussed: discussion or lack of discussion about medical contraception with others and the underlying reasons; discussion or lack of discussion with spousal partner on medical contraception and the underlying reasons and initiation of discussion of medical contraception in the couple (who should take the initiative and the underlying reasons). Interviews transcribed entirely by the author, were the subject of thematic analysis, which consists of reducing and synthesizing the transcribed material into a certain number of themes which reflects the essential, core information [22]. It should be emphasized that "the theme is a set of words making it possible to define what is discussed in the extract from the corresponding corpus while providing an indication of the content of the words" [22, p: 242]. At the end of the thematic analysis, several themes were identified highlighting the determinants of the negotiation about the use of medical contraception in the Beninese married couples. These factors are described and discussed in the next section.

## Results and discussion

### Factors influencing the negotiation of the use of medical contraception within married couples

The thematic analysis shows that three types of factors influence the negotiation of the use of medical contraception within married couples: factors that prompt spouses to discuss contraception factors that limit or hinder negotiation of the practice and the factor that plays a mixed role.

#### Incentives for negotiating the use of medical contraception within married couples

Of all the elements that influence the negotiation of the use of medical contraception, the incentive factors are the most important in the respondents’ speech. According to respondents of both sexes; contraceptive stakes, the physical impacts of multiple obstetric experiences; household resource issues and the male partner’s positive attitude in favor of contraception are the factors that encourage discussion about contraception medical care between spouses.

*Contraceptive stakes*: *family planning and the harmful effects of contraceptives*. According to respondents, contraceptive stakes refer to the possibility of limiting or spacing births and the health risks associated with the use of medical contraceptives as well as the management of the side effects of these products. Some respondents report that these stakes prompt a spouse to engage in the discussion of medical contraception with their partner. Konditamde (2017) makes the same observation in rural areas of Burkina-Faso [23]. On this point, the author indicates that despite the reluctance observed in terms of the use of contraception, men who decide to control fertility get involved in the contraceptive practice of their wives. This assumes that these men discuss contraception use with their wives. Authors report in another study conducted in Nigeria that 40% of respondents said they discussed modern contraception as a couple to negotiate the advisability of an additional pregnancy while 44% did so with the aim of stopping the use of contraception [24]. Turning to concerns about the harmful side effects of medical contraceptives and their management; other works show that some spouses resort to discussion because adverse effects [25] especially continuous bleeding, have significant psychological costs for women [26], causes problems in couples, and thus leads partners to talk about contraception [27]. These findings corroborate the comments of respondents in my research, who shed light on the role of contraceptive issues as triggers for the discussion around contraception within married couples.

“[…] In my family, there is only one person who managed to be someone… because my father died young. My mother had placed us with other families who used us for household chores, and no one had gone to school. If my mother did not have many children, she could have looked after us better. I bring this up with my husband […] to make him understand the need for birth control. When I broach the subject of contraception, my husband listens to me and that makes me very happy” [Woman, 37 years old and mother of 3 children].

“Yes, I am discussing contraception with my husband because we want to delay pregnancy. I work, and my employment contract does not allow me to get pregnant. I told him about it, and he agreed "[female, 31, mother of one].

"[…] I talk a lot about it [contraception] with my wife [..]. We talk about it when she complains of minor ailments and we wonder if that is not the root of her health problem " [Male, 45, father of 4 children].

“Yes, I discussed contraception with him [the husband] once because he said he was tired of the condom. And we agreed to use the implants” [Female, 32, mother of one].

It appears in these excerpts from interviews that the need to plan births, health concerns and discomfort associated with contraceptive use led spouses to discuss the practice. Thus, contraceptive issues are determinants of the negotiation of contraception within married couples.

*The impact of maternity on women’s body and inherent health issues*. Most of the respondents cite multiple or close pregnancies as a danger to maternal health. In clear terms, they report that multiple and/or close obstetric experiences deteriorate and weaken the health of women who fear for their lives, and that this concern motivates debate on contraception within their relationship. Note that this theme is more significant in the discourse of women. In other words, the women insisted on this theme more than the men. In addition, the use of contraception due to wear and tear on the maternal body was observed in 2005 by Bledsoe in rural Gambia [28]. According to the researcher, Gambian women living in rural areas are cautious about contraception early in their reproductive life to comply with local pronatalist norms. However, most use contraception for stopping reproduction in their 30s, because at that age, they already think they are too old to have new pregnancies. In addition, they explain their early old age by the wear and tear of the body due to many previous pregnancies. And fearing that they will die from another pregnancy, they use contraception to end childbearing. The reproductive life experiences reported by Bretin (2004) also indicate how the wear and tear of the maternal body and its health implications mobilize spouses for the discussion of contraception [29]. The reproductive trajectories drawn up by the author tell the story of several women, including Leïla and Fadéla. These two women of North African origin and living in France have had difficult reproductive paths, including early motherhood, numerous and closely spaced pregnancies, miscarriages, etc. Moreover, the seriousness of their situation appears in the remark below made to Leïla in a Maternal and Child Protection Center (MCP) about her very close pregnancies:

"This is the third, if you keep going like this you’re going to die. It’s not good to have kids every year […] plus it’s tiring” [28, p: 99].

This situation led women to discuss with their husbands the threats that further pregnancies pose to their lives. And together with their husbands, they opted for female sterilization. However, doctors ultimately refused this option and favored injectable contraceptives due to the womens ‘young age, reports the author. Multiparity and the resulting wear and tear of the maternal body, as well as the related risks of maternal death, were linked to the initiative of marital dialogue around medical contraception in my study. Here are some opinions that illustrate the phenomenon in the respondents’ comments:

"[…] The woman must talk about contraception in her marriage because she is the one who suffers the martyrdom of pregnancy. The man impregnates you and walks away. You are the woman who endures the psychological and physical challenges of pregnancy. Keeping a pregnancy to term is no easy task. The pregnancy is too crippling” [Woman, 47 years old, mother of 4 children].

"The woman should talk to her husband about contraception […], it is the woman who carries the pregnancy and its consequences. Even if a man has twenty children, he bears no mark of pregnancy, but the woman who accepts this kills herself […]. Pregnancies that are close together kill her slowly” [Male, 38, father of 3 children].

“[…] the woman must discuss contraception with her husband […], numerous pregnancies destroy a woman’s body. Her body gets tired of carrying the pregnancy all the time” [Woman, 35, mother of 5 children].

"[…] The woman should initiate the discussion about contraception because she has to be concerned about her own health. When we have too many children, we put the uterus in danger because it weakens" [Woman, 29 years old, mother of 2 children].

In these statements, the respondents present the weakening of the maternal body because of numerous pregnancies as being a legitimate reason for the dialogue on contraception within the couple. Thus, we believe that the wear and tear of the maternal body is an incentive for the discussion about contraception in the married couples.

*Household resource problems*. In their interviews, most respondents said that material and financial difficulties were at the origin of the negotiation of the use of contraception in their marriage. According to their discourse, one of the spouses initiated the discussion on contraception when the relationship between the size and the means of the household handicapped the fulfillment of parental roles. Raising the issue of contraception as a topic of marital discussion to alleviate precarious household conditions is a fact reported in other studies. In a rural area of Madagascar where Binet and Coll. (2007) analyzed fertility decline, interviewees confided that land pressure marked by the reduction in agricultural land per capita and the inability to maintain a large family mobilized the joint decision to use contraception among couples [30]. Also, in focus groups carried out in Togo as part of the study of gender relations and reproductive behavior, illiterate city dwellers declared that the negotiation of contraceptive use in their couples was motivated by economic precariousness [31]. This attitude is reminiscent of what Cosío-Zavala (1999) called the Malthusianism of poverty to designate the planning of births by modest Latin American couples as a means of resilience in the face of poverty [32]. The following excerpts from the discourse highlight the same attitude among the people we surveyed.

“Yes, my husband and I had discussed it [contraception] when we think our children are getting too many. I initiated this discussion because our means could no longer meet our needs. And I thought about finding a way to stop the pregnancies "[Female, 55, mother of 8].

“My husband too is aware of the gravity of the situation and when I brought up the stakes of contraception, he was open to my message. It was after our misfortune with the last pregnancy which cost us our economy and caused us debts before the child finally died that I decided to discuss the subject of contraception with my husband” [Woman, 28 years old, mother of 6 children].

"I talk about contraception with my wife because […] I am the one who takes out the money to pay for the care. Yesterday, for example, after returning from the prenatal consultation, she presented me with a prescription for over 15,000 francs that I had to pay immediately. It is already painful for me to pay this amount "[Male, 44 years old, father of 4 children].

On the other hand, it should be emphasized, as noted in these statements, that respondents of both sexes are concerned about the use of contraception as a strategy to mitigate the material and financial problems of households. However, men who say they have discussed contraception as a couple do so because they are having difficulty financing the costs of maintaining their homes. In contrast, women tend to negotiate contraception because they experience the reality of insufficient household resources more acutely than their male partners. This is because, in the Beninese context, they are culturally responsible for the tasks of care and social education of children that men are called upon to finance. As such, they are the ones who manage the insufficient resources of the household daily to ensure the continuity of parental functions. It is for this reason that they are more sensitive than their spouses to resource problems and put them forward in the negotiation of contraception as a couple. In view of the above, it seems that household resource difficulties are an important determinant of the discussion of the use of contraceptive practice by married couples.

*Husband’s approval of medical contraception*. The positive attitude of men towards contraception was also mentioned as a factor facilitating the negotiation of the practice by the couple. This point was raised by women who say they have started negotiating contraception usage as a couple because their husbands have a positive perception of it. Indeed, contraceptive use is essentially a couple’s business in developing regions [33]. Nevertheless, studies often show a strong heterogeneity of contraceptive demand in couples because women are more interested in the use of contraception than their husbands [34]. In addition, in developing regions, contraceptive use is influenced by gender relations in which men act as decision-making authority and provide the means to acquire contraception [35]. These data suggest that the ability to predict the attitudes of their husbands towards contraception matters for women in developing regions who wish to discuss the practice as a couple. In this regard, Lasee and Becker (1997) found that in Kenya, 67% of married women and 75% of men correctly predicted the positive attitude of their spouses towards contraception [36]. In other words, a lot of married people know their partners’ position on contraception. This competence allows women wishing to negotiate contraception in their marriage to do so with more success. Also, women who correctly predicted the approval of the practice by their husbands were able to discuss it with them, as seen in the statements below.

“Yes, I talk about contraception with my husband because he likes it. But I must change my method […].” [Woman, 32 years old, mother of 5 children]

“I talk about contraception with my husband because my husband likes it” [Female, 23, mother of one].

From the above, it can be said that the positive attitude of men towards contraception makes it easier to negotiate the practice in their relationship. Apart from these elements that encourages negotiation of contraceptive use by married couples, respondents highlighted other factors that we will discuss in the next section.

#### The factors limiting the negotiation of the use of medical contraception within married couples

According to some respondents, two factors hinder the couple’s dialogue around medical contraception: the disinterest of one of the spouses for the practice and the timidity of the woman in the couple.

*The lack of interest of one of the spouses in medical contraception*. For various reasons, many people show a lack of interest in contraceptive use in developing countries [37]. In Benin, for example, the low contraceptive prevalence can be explained among other things by social, marital, or individual disapproval of medical contraception [38]. Thus, the disapproval of contraceptive use by a married person leads to disinterest which makes it difficult to discuss medical contraception with the marital partner. On the other hand, people, especially women who have had the opportunity to increase their interest in contraception are more inclined to discuss it as a couple. For example, a study of the extramarital determinants of marital communication in matters of family planning shows that Kenyans with a weak social network are less tempted to talk about contraception as a couple, while those who work in voluntary organizations discuss it better with their spouses [39]. For that, the author points out that women with fewer social networks would miss opportunities to discuss contraception with others. It is interesting to examine the comments below reported by one respondent reflect the influence of the lack of interest in medical contraception on the discussion around this practice by the couple:

“[…] My husband and I never talked about medical contraception. I don’t want to talk to him about it because I’m not interested "] Female, 29, mother of 3].

All in all, a lack of interest in contraception turns out to be a detrimental factor in the couple’s discussion about contraception. Thus, this phenomenon constitutes an obstacle to the negotiation of the practice between spouses.

*Married women’s shyness within the relationship*. It can happen in marital dynamics that one of the partners, is shy. Such a posture could interfere with marital negotiation of contraceptive use. In principle, the shyness of the man and / or his partner can hinder the couple’s debate on contraceptives. Nevertheless, in many respects, the discursive material suggests that it is the timidity of women in relationships that inhibits the discussion. Almost all the respondents agree on the principle that the woman is the member of the couple who should initiate the debate on contraception. The arguments supporting this vision flourish in the discourse and touch on issues of maternal health. Respondents believed that children benefit the couple while it is the woman alone who bears the stigma and the after-effects of obstetric experiences. For this, she must initiate the discussion on contraception in the couple to preserve her own health, particularly because demographic and health surveys in developing countries continuously show that men are more birth-prone than their wives resulting in lukewarm attitudes towards family planning. In her work examining the process of contraceptive appropriation by men, Desjeux (2012) finds that couples’ contraception is initiated by women who educate their partners and prescribe specific practices [40]. In short, a woman’s shyness in marital relations harms the couple’s dynamics in contraceptive matters, as can be read in the words of this respondent.

“[…] Some women cannot talk about contraception as a couple. If the woman is shy, it will not work for the couple” [Woman, 39 years old, mother of 4 children].

In view of this observation, we can estimate that the timidity of the woman in the marital dynamics is an obstacle to the negotiation of the contraceptive use between the spouses.

#### Factor with a mixed or ambivalent impact in negotiating the use of medical contraceptives within married couples

*Reproductive goals or intention of fertility*. Reproductive goals were also mentioned as an element that influences the discussion of contraception in married couples. According to the respondent who reported this element, it plays a mixed role in the marital negotiation of the practice. In fact, the respondent emphasized that a reproductive goal–that is to say, the number and quality of children wanted by one of the spouses–is decisive in initiating the marital debate about medical contraception. For good reasons, spouses sometimes pursue divergent reproductive goals [33] which have a negative impact on their relationship to contraception. For example, a study carried out among Ghanaian couples indicates that 17% disagree on the possibility of having another child and, in two out of three cases where the man wanted one more child, his wife did not. The author also specifies that because of the unequal power relations between the sexes in Ghana and the desire to avoid conflicts, the concordance between the reproductive objectives of the spouses can mask a prior agreement of the couple on the subject. This observation suggests that the extent of spousal disagreement over fertility goals is greater than it appears in this study. Such a situation influences the negotiation and contraceptive use of the spouses according to the results of Dodoo (1993) and Barden-O and Speizer (2010) [41, 42]. On the other hand, Barden-O and Speizer (2010) and Hossain and Coll (2007) have shown that couples who disagree about the desire for an additional child use contraception less than those were both members want to limit births [42, 43]. This was observed in the comments of a respondent who answered the question of which member of the couple could take the initiative in the debate around contraception and the reasons justifying their choice. Here is an excerpt from his statement:

" It depends. Both can initiate because if the reproductive goal of the number and sex of children is not achieved for a member of the couple, then this person object to contraception and does not want to talk about it. This is valid for both men and women. On the other hand, once satisfied, the person can open the debate on contraception "[Female, 31, mother of a daughter].

All in all, the discordance of reproductive objectives influences fertility practices, including the conjugal discussion of contraception. In clear terms, a reproductive goal may encourage or hinder marital dialogue about contraception. Therefore, this factor is an integral part of the determinants of the negotiation of contraception in the married couples.

## Conclusion

Medical contraception is arguably one of the greatest inventions of the human mind because of the major role it plays in human development. However, millions of married women still fail to access and use this technology for various reasons [6, 41–43]. Most of the women concerned live in developing regions [44] where negotiation of contraception within the couple is essential before women will practice it [13–15, 33]. Hence the importance of identifying the factors that influence the negotiation of the use of contraception within married couples.

The results of this study show that contraceptive stakes, the impact of multiple obstetric experiences on women’s body, household resource problems and the male partner’s positive attitude in favor of contraception lead married couples to negotiate contraception. On the other hand, the disinterest of one of the spouses towards medical contraception and women’s timidity in marital dynamics constitute obstacles to the negotiation of the practice between spouses. In addition to these factors, there is the reproductive goal or the intention of fertility, which plays a mixed role in that it becomes, depending on the case, a lever, or an obstacle to discussing contraception within married couples.

It should be noted that the most important discovery of this study relates to the fact that respondents unanimously agreed with the idea that it is the woman who should take the initiative to negotiate contraception in the couple because of her relationship to motherhood and the resulting health implications. This is crucial information for family planning service providers in Benin where the power of fertility decision-making rests with men who are lukewarm supporters of contraception because they want more children than their wives. Since both women and men report in this study that it is in the interest and responsibility of women to initiate the debate on medical contraception use within married couples; family planning service providers i.e., nurses, midwives and community health workers should support women who wish to limit or space births with medical contraception. To do this, they must encourage them to discuss it with their husbands because men themselves recognize that it is important for women to initiate the discussion on contraception in the couple. This coaching would be based on the influencing factors of negotiating the use of medical contraception within married couples and the personal life experience of each woman to serve her in her best interests.

## Supporting information

S1 Annex (DOCX)

S2 Annex (DOCX)
