# Supplementary material for: Negotiation of the use of medical contraception: Levers and obstacles within married couples in Benin
Source: PLoS One. 2021 Jul 22;16(7):e0253438. doi: 10.1371/journal.pone.0253438 (PMC8297886; doi:10.1371/journal.pone.0253438)
Supplement: S1 Annex — (DOCX) [file pone.0253438.s001.docx]

S1 Annex

SOCIO-DEMOGRAPHIC DATA FORM

Questionnaire to be completed by the respondent or administered to him / her.

Important notice: Please note that your answers will be kept strictly confidential!

1. Gender: 1. Male *o* 2. Female *o*
2. Place of residence
3. Name of the city or village: ………………………………………. (Specify name)
4. Name of Neighborhood: ……………………………… (Specify name)
5. Religion

Check only one answer among the following!

1. Christianity *o*
2. Islam *o*
3. Traditional religion *o*
4. Atheism *o*
5. Others *o* ……………… ......... (specify religion)
6. What job do you do for a living? ……………………………………
7. What is the job / job of your spouse? …………………………………
8. What is your level of education?

Check only one answer among the following:

1. Never attended school *o*
2. Primary *o*
3. Secondary *o*
4. Higher education (University or others...) *o*
5. What is your spouse's level of education?

Check only one answer among the following:

1. Never attended school *o*
2. Primary *o*
3. Secondary *o*
4. Higher education (University or others...) *o*
5. How old are you? .......................................... ............ (Specify age)
6. How old is your spouse ..................? (Specify the age)
7. How long have you been living as a couple with your partner? ............. (Specify the year)
8. What type of union do you live in?

Check one answer.

1. Monogamous union o
2. Polygamous union o
3. Before your marriage, did you wish to have any child (ren) in your life?

1. Yes*o* 2. No*o*

1. How many children would you like to have? .................... (Specify the number)
2. Specify the number of children of each sex you want.
3. Number of girls: ………………………… (Specify the number)
4. Number of boys: …………………… .... (Specify the number)
5. Having children according to providence (God’s will) *o*
6. How many children do you have now? ……………… (specify the number)

Specify how many children of each sex you have.

1. Number of girls: …………………………. (Specify number)
2. Number of boys: …………………… ...... (Specify the number)
3. At what age did you have your first child? .............................
4. Are you pregnant or is your partner pregnant? ...

Check one answer 1. Yes *o* 2. No *o*

1. If yes, is your current pregnancy desired? ........ 1. Yes *o* 2. No*o*
2. If no, was your last pregnancy desired? ... 1. Yes*o* 2. No*o*
