# Supplementary material for: Negotiation of the use of medical contraception: Levers and obstacles within married couples in Benin
Source: PLoS One. 2021 Jul 22;16(7):e0253438. doi: 10.1371/journal.pone.0253438 (PMC8297886; doi:10.1371/journal.pone.0253438)
Supplement: S2 Annex — (DOCX) [file pone.0253438.s002.docx]

S2 Annex

Theme: Conjugal discussion on medical contraception

1. *Have you ever discussed contraception with others? ............. 1. Yes o 2. No o*
   1. *If so, who did you talk to and why?*
   2. *If not, why don't you discuss contraception with other people?*
2. *Have you ever discussed contraception with your partner…? 1. Yes o 2. No o*
   1. *If so, why, and how did you come to this discussion? How did this discussion go? Are you satisfied with the outcome of any discussions you have about spacing or avoiding pregnancies?*
   2. *If not, why?*
3. *In your opinion, who can initiate the discussion on contraception in a couple and why?*
4. *Do you have any other things to say about the discussion of contraception between spouses that we have not covered in this interview?*
